# Delphi Consensus Recommendations for the Development of the Emergency Medicine Point of Care Ultrasound (POCUS) Curriculum in Nepal 

**DOI:** 10.24908/pocus.v9i2.17724

**Published:** 2024-11-15

**Authors:** Anmol P Shrestha, Wolfgang Blank, Ursula H Blank, Rudolf Horn, Susane Morf, Sanu K Shrestha, Shailesh P Shrestha, Samjhana Basnet, Anjana Dongol, Raj Kumar Dangal, Roshana Shrestha

**Affiliations:** 1 Department of General Practice and Emergency Medicine, Dhulikhel Hospital, Kathmandu University School of Medical Sciences Dhulikhel NPL; 2 University of Tübingen, German societies of ultrasound in Medicine (DEGUM) Tübingen, DEU; 3 German societies of ultrasound in Medicine (DEGUM) DEU; 4 Swiss Societies of Ultrasound in Medicine (SGUM) Mustair CHE; 5 Department of Obstetrics and Gynecology, Dhulikhel Hospital, Kathmandu University School of Medical Sciences Dhulikhel NPL

**Keywords:** Curriculum Development, Emergency Medicine, Point-of-Care Ultrasound (POCUS), Lung Ultrasound, Focused Cardiac Ultrasound

## Abstract

**Introduction: **Emergency Medicine Point of Care Ultrasound (EM-POCUS) is a diagnostic bedside tool for quick and accurate clinical decision-making. Comprehensive training in POCUS is a mandatory part of EM training in developed countries. In Nepal, we need to build an educational curriculum based on the local medical system, available resources, and educational environment. We used the modified Delphi method to develop a EM-POCUS curriculum. **Methods: **We formed an EM-POCUS core working group based on expertise in key identified areas. The core working group developed criteria for expert panelist selection and synthesized the data for panelists after each Delphi round. We recruited 46 expert panelists to participate in a series of electronic surveys. The literature review and the results of the first Delphi round identified a set of competencies. Quantitative methodology was performed for subsequent surveys. Data analysis of the frequency, percentage, median, and interquartile range of the 9-point Likert scale was performed. We deemed a minimum threshold of 80% agreement to retain items across Delphi rounds. The result of every round was disseminated before subsequent rounds for the expert panelists to review responses in light of the group’s response. **Results**: We identified 10 specific global competency categories and 132 objectives (Round 1, response rate 85%). Rounds 2 and 3 (response rates 78% and 81% respectively) developed consensus on 45 core objectives (34%). The list of EM-POCUS competencies with the median (IQR) was finalized. **Conclusion: **This expert, consensus-generated EM-POCUS curriculum provides detailed guidance for EM-POCUS education and applications in clinical practice in Nepal.

## Introduction

Point of care ultrasound (POCUS) has become standard practice in emergency departments (EDs), ranging from remote rural hospitals to well-resourced academic centers. Emergency Medicine (EM)-POCUS, performed by the treating clinician, comprises a set of focused ultrasound applications to facilitate early diagnosis, guide invasive procedures, and inform safe management and timely disposition decisions for patients 1. One of the several advantages of incorporating EM-POCUS in daily clinical practice is an integration of sonographic findings with history and clinical examination at the patient's bedside. Prior research demonstrates that EM-POCUS improves patient safety, diagnostic reasoning, and certainty with immediate results 2, 3, 4. Moreover, in resource-limited settings, ultrasound is an effective means of cost reduction 5. The EM-POCUS examination may range from a goal-directed tool with limited scope to a comprehensive and standard consultative examination depending upon the clinical situation 6.

The American College of Emergency Medicine (ACEP) position paper 7 and a similar document by the Society for Academic Emergency Medicine in 1991 8 supported the use of EM-POCUS. With this support, EM residency programs in the United States and Canada introduced ultrasonography as a standard part of training 9, 10. The emergency ultrasound guidelines developed by ACEP^10^ described the scope of practice for EM-POCUS to include seven ultrasound competencies: trauma, pregnancy, abdominal aorta, cardiac, biliary, urinary tract, and procedural. These were further expanded in 2009 to include thoracic, deep vein thrombosis (DVT), ocular, and soft tissue/musculoskeletal 11. A consensus recommendation was published by the ACEP in 2009 which served as a POCUS training guide for all EM residencies 12. In 2011, the ACEP developed guidelines for fellowship training 13 addressing dedicated ultrasonography practice, education, or research. Likewise, EM-POCUS is used in many hospitals in Europe 14. Worldwide, there are a large number of clinical studies, systematic reviews, and consensus papers by experts and professional societies that form the basis for the many recommendations for the entire range of EM-POCUS in the ED 1, 2, 3, 4, 5, 6, 7, 8, 9, 10, 11, 12, 13, 14, 15, 16, 17, 18, 19. There is also evidence that EM-POCUS speeds up consultation processes 2, 20. However, these cannot be directly implemented in resource-limited countries like Nepal, where EM is comparatively a young clinical domain. The type of core EM-POCUS application depends on local needs, resources, and practice patterns, which can vary significantly across regions and countries. Likewise, the disease prevalence, the impact of the disease, the potential for patient benefit, and resources should be used to guide what core applications are considered 21. The classification of applications as core versus advanced is also evolving at a rapid rate 22. 

EM is one of the youngest recognized specialties in Nepal, and its growth in clinical practice and academic development has been challenging. In Nepal, several EM training modules and programs are currently practiced which are fragmented with different curricula and durations 23. General Practice post graduate doctors have been providing emergency care services for the country since 1982. Furthermore, EM in Nepal has evolved, with various other programs now available in addition to Doctor of Medicine (MD) in General Practice and Emergency Medicine (GPEM) to become EM physician. These include EM fellowship, MD EM, and Doctorate of Medicine (DM) in EM. This may have contributed to the different levels of ultrasound training emergency physicians (EPs) receive. Moreover, there are varying responsibilities of EDs in urban and rural settings. There is no nationally recognized curriculum in EM-POCUS for Nepal. Comprehensive training in POCUS is currently a mandatory part of EM training in developed countries.

In contrast to the western setting, there had been limited effort to expand this knowledge and skills in Low-Middle Income Countries (LMICs) in the past 24. However, in recent times, the use of EM-POCUS has increased in clinical practice in LMICs, including Nepal, as supported by the increasing evidence supporting various EM-POCUS applications 25, 26, 27, 28, 29, 30. There are limited publications related to educational interventions to enhance POCUS in the ED in Nepal 31. 

Since 2017, Dhulikhel Hospital-Kathmandu University School of Medical Sciences (DH-KUSMS) has been conducting a two-day interdisciplinary emergency ultrasound workshop to provide quality continuing education programs in collaboration with German (DEGUM) and Swiss (SGUM) societies of ultrasound in Medicine. It is a systematic training using an integrated teaching approach of lecture, assigned reading, testing, demonstration, and clinical scanning of models. Despite this requirement, training guidelines regarding EM-POCUS knowledge and skills have yet to be developed in Nepal. Our study attempts to bring EPs from different backgrounds together to develop a curriculum and a competency checklist for POCUS education in Nepal using the modified Delphi consensus method. This was one of the first concrete steps towards consolidating and building up a consensus curriculum based on local expertise, availability of resources, and local needs. 

## Methods

Panelists

This study had two sets of panelists: the EM-POCUS core working group and the EM-POCUS expert panelist group. There was a deliberate effort to recruit experts and educators from most postgraduate training sites, both public and private, that represented all academic streams (MD GPEM, Fellow EM, DM EM).

A team of 17 physicians fulfilling the criteria served as the core working group for this project. An initial meeting was organized at the Dhulikhel Hospital (DH) which served as a starting point for this collaborative effort to gather and refine expert opinions. A shared online workspace with this group was established on Google Drive to share relevant literature on existing EM-POCUS guidelines, curricula, and evidence supporting current applications. This team developed criteria for Delphi expert panelist selection, synthesized and reported the data to panelists after each Delphi round, and determined when the Delphi process was completed. From a curriculum standpoint, the core working group categorized data and processed panelist contributions into a structured format to create the final checklist of core objectives and competencies.

The definition of “expert” was agreed upon by the study authors. We decided on the panel size as per recommendations by Hsu and Sandford 32. The 46 expert panelists fulfilling the criteria (Table 1) were identified using a purposive and snowballing sampling technique. To capitalize on collective expertise, some members (n=13) of the core working group also served as panelists on the Delphi expert panel (n=46). The panelists neither had interaction nor did they know individual panelists’ opinions which assured their anonymity 33. This allowed all panelists’ responses to be equally weighted. The feedback was facilitated by the core working group which allowed each panelist to review their response in light of the group’s response. 

**Table 1:** Characteristics of the EM-POCUS core working group and expert panelist group

**A**	**EM-POCUS core working group (ONE of the following criteria)**
1	Physicians who had completed MDGPEM and/or Fellow EM and/or DM EM, had more than 3 years of work experience in the ED and were actively using POCUS in their workplace.
2	Involved in curriculum development in their respective institution for MD GPEM, fellow EM, or DM EM.
3	National and international faculty involved in EM POCUS education training in Nepal.
**B**	**EM- POCUS expert panelist group: (ONE of the following criteria)**
1	Physicians who had completed MDGPEM, with or without EM fellowship/doctorate, and physicians who had MD EM degree with more than 2 years of work experience in the ED and practice POCUS in day-to-day clinical practice
2	An EM-POCUS training graduate from DH-KUSMS, currently practicing POCUS in clinical practice.
3	National and International faculty involved in POCUS training at DH-KUSMS or have worked in Nepal ED who know the local context and needs.

EM-POCUS= Emergency Medicine- Point of Care Ultrasound, MDGPEM= Masters in General Practice and Emergency Medicine, Fellow EM=Fellowship in Emergency Medicine, DM EM= Doctorate of Medicine in Emergency Medicine, ED=Emergency Department, DH-KUSMS= Dhulikhel Hospital-Kathmandu University School of Medical Sciences

Study design and procedure

We utilized the modified electronic Delphi (e-Delphi) method during the survey. For this purpose, we used the Kobo Collect and Kobo Toolbox applications 34. Delphi is a method that structures communication into an effective process allowing content experts to confront and solve complex problems 35, 36, 37, 38. For our study, we modified the conventional Delphi into an electronic version (e-Delphi) that utilized email and electronic survey platforms. The design is based on recommendations by Witkin and Altschuld 39. The content was generated by the expert panelists with up to three iterative rounds. The anonymity of panelists’ contributions was maintained. The reporting for this study adheres to the CREDES (Guidance on Conducting and Reporting Delphi Studies) guidelines 40. (S1)

Round one survey

Qualitative methodology was used for the e-Delphi process for the first round. An open-ended questionnaire with three open questions was circulated among the expert panelists. The questions included:

In your opinion, what core knowledge should EM-POCUS trainees acquire to effectively use EM-POCUS as a diagnostic tool in the acute care setting? 

In your opinion, what core skills should EM-POCUS trainees acquire to effectively use EM-POCUS as a diagnostic tool in the acute care setting?

In your opinion, what behavioral impact should POCUS trainees have after completion of the training?

We sent regular “e-reminders” to encourage panelists’ completion of tasks. We revised, coded, and grouped the answers to determine the list of objectives for various domains of EM-POCUS. We also conducted a literature review of different standard existing international emergency ultrasound curricula to aid the formulation of the list of objectives 8, 9, 10, 21. The EM-POCUS core working group reviewed and revised the questionnaires for face and content validity before dissemination to the expert panelists for subsequent surveys.

Round two and subsequent surveys

We utilized quantitative methodology for subsequent rounds of surveys. The expert panelists evaluated the importance of listed knowledge and skills objectives on a 9-point Likert rating scale where scores 1-3, 4-6, and 7-9 represented non-essential, optional/elective/advanced, and essential/critical/core objectives, respectively. If the panelists could not rate the objective, they were requested to choose option 0 and elaborate on the reasons in the textbox at the end of each section. The core competency objectives are a necessary component of the required service delivery and all EPs should be able to perform this. The non-essential competency objectives are inappropriate for EPs and should not be performed at that level. The optional, elective, or advanced competency objectives may be appropriate in some selected contexts, but not a requirement for all EPs.

**Figure 1:**
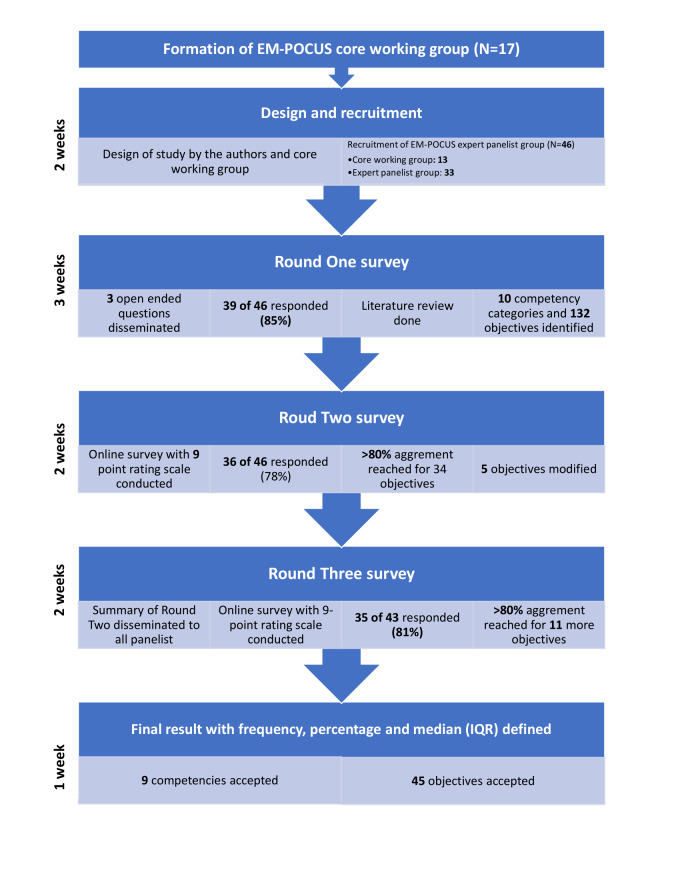
The stages of Delphi process and outcomes.

We deemed a minimum threshold of 80% agreement to retain objectives across Delphi rounds utilizing cascaded consensus-related termination criteria. The 80% cutoff is based on Lynn’s suggestion that at least 80% of experts must agree on an objective to achieve content validity when at least ten experts participate in consensus development 41, 42. We calculated and disseminated the medians with the interquartile range (IQR) for those components that did not reach consensus for consideration in subsequent surveys 36. The median value represented the middle value of the responses, giving a measure of central tendency. The IQR provided an idea about the dispersion of the responses. Together, they offer a comprehensive understanding of the consensus and variability within the expert opinions in the survey. We also recommended panelists to provide additional comments in each round which allowed the panelists to guide the responses from fellow panelists in successive rounds. We provided feedback that included frequencies, percentages, median with IQR, and comments related to objectives from round two to the panelists. The panelists then voted on objectives that received less than 80% agreement. Between rounds, we analyzed the data and modified the survey questionnaire to include the data and comments. Some objectives were discarded and we presented the modified survey to the panelists for consideration in the subsequent round. The procedure is summarized in Figure 1. 

Ethical clearance

This study was reviewed by the KUSMS Institutional Review Committee (IRC approval number 32/24 on 26 January 2024). Informed written consent was obtained from the core working group. Before consenting for the Delphi survey, a detailed explanation regarding the process and anonymity to maintain the privacy and confidentiality of the expert panelists was provided. We also included the option to opt out from completing the survey form before each survey round. 

## Results

The final Delphi panel of 46 included the 13 core working group panelists and an additional 33 expert panelists. The demographic profile of the final panelists is summarized in Table 2.

**Table 2:** Demographic profile (frequencies and percentages) of EM-POCUS Delphi expert panelists (N=46).

**Variables**	**N(%)**
Sex (Male)	32 (69.6)
**Practice Setting **	
Medical College	26 (56.5)
Hospital setting	12 (26.1)
International	8 (17.4)
**Location **	
Kathmandu (capital)	22 (47.8)
Outside Kathmandu	16 (34.8)
International	8 (17.4)
**Educational Background **	
MD General Practice and Emergency Medicine (MDGPEM)	21 (45.7)
MD Emergency Medicine	8 (17.4)
MD GPEM + Fellow Emergency Medicine	8 (17.4)
MDGPEM + DM Emergency Medicine	5 (10.9)
MD Radiology	2 (4.3)
Others	2 (4.3)
**Frequency of Use of Ultrasound in Clinical Setting (N=36) **	
Often	17(47.2)
Always	11(30.6)
Sometimes	8(22.2)
**Work experience after post-graduation (N=36) **	
>5 years	32 (88.9)
3-5 years	2 (5.6)
<3 years	2 (5.5)

In round one, 39 out of 46 panelists (85%) completed the survey. Based on the result of the round one survey, and extensive literature review, we identified ten specific global competency domains (Table 3).

**Table 3:** List of global competencies

**SN**	**Code**	**Competency domains**	**Number of objectives**	**Number and % of objectives reaching consensus (≥80%)**
1	upk	Ultrasound physics and knobology	9	7 (78%)
2	lus	Lung ultrasound and airway	13	8 (62%)
3	focus	FOCUS	15	7 (47%)
4	v	Vessels	11	3 (27)
5a	hsp	Abdominal ultrasound (Liver, spleen, pancreas)	16	5 (31)
5b	kub	Abdominal ultrasound (Kidneys, bladder, prostate)	20	3 (15%)
6	ob	Obstetric and gynecological ultrasound	18	5 (28%)
7	msk	Musculocutaneous ultrasound	8	1 (13%)
8	oc	Ocular ultrasound	5	0 (0)
9	pdr	Procedural ultrasound	11	4 (36%)
10	pcl	POCUS based protocols	6	2 (33%)
		Total	132	45 (34%)

The response rate for the second round of the survey was 78% (36/46). Two panelists withdrew voluntarily, and one response was excluded as all the objectives were rated on one scale. Panelists reached the 80% consensus threshold for 45 core objectives (Table 4). The median with IQR was calculated for all objectives. Five objectives were modified according to the proposed comments by the panelists. Before the third round of the survey, we provided findings on the performance of competencies (frequency, percentage, median with IQR, and any comments by panelists) from round two to the expert panelists. The response rate for round three was 81% (35/43), and consensus on 11 more objectives was achieved. Although the median was not considered as a criterion to determine consensus, all objectives that reached >80% had median value and IQR within the consensus category of 7-9. The list of final EM-POCUS competency domains with frequency, percentage, and median (IQR) is defined and presented (Supplemental file 2). The final compilation of objectives was shared and discussed with the core working group and validated in an online platform before publication.

**Table 4:** The final result of the Delphi panel: a list of global objectives reaching a consensus of ≥ 80%.

**SN**	**POCUS Objectives **	**Core%**	**Advanced %**	**Not needed %**	**Median score**	**IQR**
1	Describe the basic ultrasound modes: B-mode, M-mode, and doppler.	87.9	12.1	0	8(7-9)	2
2	Choose the appropriate probe type for various applications	93.3	6.7	0	9(8-9)	1
3	Describe the various probe orientations and standard scan planes	90.9	9.1	0	9(8-9)	1
4	Demonstrate the use of ultrasound controls and knobs for image optimization	84.8	15.2	0	8(8-9)	1
5	Interpret common ultrasound artifacts	81.8	18.2	0	8(7-9)	2
6	Describe the sonographic characteristics of structures with varied densities	84.8	15.2	0	8(7-9)	2
7	Describe the infection prevention controls when using an ultrasound machine*	91.2	8.8	0	8(8-9)	1
8	Demonstrate normal lung sliding	97.0	3	0	9(8-9)	1
9	Demonstrate normal lung pulse	81.8	12.1	6.1	8(7-9)	2
10	Enumerate the causes of absent lung sliding	93.9	6.1	0	8(7-9)	2
11	Demonstrate M-mode to elicit sea-shore or bar-code sign	84.8	12.2	3	9(8-9)	1
12	Elicit the lung point sign to confirm pneumothorax	81.8	18.2	0	8(7-9)	2
13	Recognize free fluid in the pleural space	93.9	6.1	0	9(8-9)	1
14	Demonstrate LUS signs of lung consolidation*	80.0	20	0	7.5(7-8)	1
15	Demonstrate LUS signs of interstitial syndrome*	82.3	11.8	2.9	8(7-9)	1.75
16	Acquire and Interpret ultrasound images of the heart in the 4 standard ultrasound acoustic windows	87.9	12.1	0	8(7-9)	2
17	Recognize the anatomy of the normal heart in the standard views	87.9	12.1	0	8(7-9)	2
18	Identify cardiac standstill	84.8	9.1	6.1	9(7-9)	2
19	Assess the global left ventricular function with visual estimation	81.8	15.2	3	8(7-9)	2
20	Assess for presence of pericardial effusion	90.9	9.1	0	9(8-9)	1
21	Recognize signs of cardiac tamponade	81.8	18.2	0	8(7-9)	2
22	Measure IVC diameter in B and M modes	93.9	3.1	3	8(7-9)	2
23	Identify the abdominal aorta*	88.2	11.8	0	8(7-9)	2
24	Measure the aortic diameter.*	80.0	20	0	7(7-8)	1
25	Demonstrate the 2-point (inguinal and popliteal) compression techniques to identify DVT of the lower extremity*	94.1	5.9	0	8(7-9)	2
26	Identify free fluid in the abdominal cavity	97.0	3	0	9(8-9)	1
27	Acquire and Interpret ultrasound images of the normal USG appearance of the Liver*	80.0	17.1	2.9	8(7-9)	2
28	Acquire and Interpret ultrasound images of the normal USG appearance of the gallbladder*	82.4	11.8	5.8	7.5(7-9)	2
29	Identify gallbladder stone	81.8	15.2	3	8(7-8)	1
30	Recognize the USG signs of cholecystitis*	80.0	17.1	2.9	7(7-8)	1
31	Acquire and Interpret ultrasound images of the normal USG appearance of the kidneys ·*	82.4	14.7	2.9	8(7-9)	2
32	Recognize hydronephrosis	81.8	18.2	0	8(7-9)	2
33	Acquire and Interpret ultrasound images of the normal USG appearance of the urinary bladder	84.8	15.2	0	8(7-8)	1
34	Assess the nonpregnant and pregnant uterus by ultrasound using an abdominal probe.	93.9	6.1	0	8(8-9)	1
35	Identify normal intrauterine pregnancy in the first trimester.	81.8	18.2	0	8(7-9)	2
36	Identify and document fetal cardiac activity	84.8	12.2	3	8(8-9)	1
37	Demonstrate the localization of the placenta in the third trimester.	84.8	12.2	3	8(7-9)	2
38	Identify free fluid in the peritoneum and utero-vesical space	87.9	12.1	0	8(7-9)	2
39	Identify subcutaneous abscess*	80.0	20	0	8(7-9)	2
40	Perform ultrasound-guided pericardiocentesis	81.8	15.2	3	7(6-8)	2
41	Perform ultrasound-guided peritoneal fluid tapping	87.9	9.1	3	9(8-9)	1
42	Perform ultrasound-guided pleural fluid tapping	84.8	12.2	3	9(8-9)	1
43	Perform ultrasound-guided intravenous cannulation	81.8	15.2	3	8(7-9)	2
44	Apply E-FAST to trauma patients	90.9	9.1	0	9(8-9)	1
45	Apply RUSH protocol to guide management decisions in patients with undifferentiated shock.	84.8	12.2	3	8(7-9)	2

* Consensus in the third round

## Discussion

The Delphi consensus protocol is the first of its kind that outlines the core content for the EM-POCUS training objectives from which we can build EM-POCUS curriculum for the Nepali context. This proposed EM-POCUS curriculum lays a strong foundation to guide the EPs to be proficient POCUS practitioners and for subsequent quality, uniformity, and growth of EM-POCUS training programs in Nepal. Through this guide, the POCUS educators in Nepal can create a POCUS program and train future POCUS leaders through curriculum development, workshops, and immersive training. 

POCUS has been widely used in many disciplines as a rapid diagnostic tool, to aid the diagnosis of multiple medical conditions ranging from acute appendicitis, airway compromise, abdominal aortic aneurysm, traumatic injury assessment, and other applications 15, 16. The relatively fast use has made it a potential option in situations where a formal radiological investigation may delay the diagnosis. However, the ever-increasing demands of other diagnostic imaging and interventional radiological procedures have underscored the importance of non-radiologist physicians' contribution to radiological diagnosis through POCUS 43.The standard consultative ultrasonography involves a delay in the performance of the study, a delay in its interpretation, and a delay in the transmission of the results to the clinical team. Therefore, improving the competencies of EPs through standard POCUS training is essential for quicker diagnosis and treatment of ED patients. 

Some unique features of the process of the study were the diversity of the expert panel, the high response rate, and the rigorous process of consensus. Although no group of experts can represent the perspective of every practicing clinician, our panelists were EPs representing all backgrounds in Nepal, working in academic and hospital-based institutions, and were based in urban and suburban regions. The panel also included international clinicians who were trained and working in developed countries but were aware of the context of the position of EM-POCUS in Nepal. Radiologists and non-EP consultants were also included as panelists to establish more consensus. Thus, the survey demonstrated inclusivity, and the results apply to Nepali EDs. 

The varied use of POCUS by EPs reflects a broad variety of responses by the panelists. This is evident in the depth and breadth of the skill sets related to competency categories that were deemed essential or core; e.g., Lung ultrasound (LUS) (8 out of 13; 62%), Focused cardiac ultrasound (FOCUS) (7 out of 15; 47%), Abdominal ultrasound (5 out of 16; 13%). The reason for such variation can be due to the special interest and working environment of EPs, as well as the different pathways and training of POCUS. The strongest agreement was for ultrasound physics and knobology (7 out of 9; 78%). The high number of LUS and FOCUS skill set objectives that were stated as core or essential reflected the global trend of use of POCUS for undifferentiated dyspnea and shock 2, 4. In contrast, most skill set objectives for ocular and musculoskeletal competency categories did not reach a consensus. This may reflect the specialized nature of these domains, less utility in practice, and the perceived need among the panelists for referral in contrast to the guidelines from the developed world 9, 10, 17.

Additionally, the working core group identified the median (IQR) for all EM-POCUS applications that did not reach 80% agreement. The authors propose that the final list of objectives for EM-POCUS curriculum will be shared with the General Practice and Emergency Medicine Association of Nepal (GPEMAN) for external validation and further academic dissemination. This may guide the POCUS educators to visualize the central tendency, range, and extent of variation in responses from the panelists to select applications that may have a positive impact on patient care and ED flow. While it is likely that each institution and program has the authority to determine which, if any, additional applications can be taught on an elective basis, we recommend that the proposed curriculum be implemented as a minimum standard for all the EM training streams and all EDs in Nepal. However, because of a lack of consensus on the remaining 87 items, corrections and supplementations will be needed in the future. POCUS is in a state of rapid evolution, therefore, there is a need for regularly scheduled curriculum reviews and updates according to the feedback from the implementation of this curriculum. 

## Limitations

The limitations of our study relate to those inherent to any modified Delphi study that relies on panelists with limited or nonexistent face-to-face contact. Although we enrolled expert panelists based on their achievements in the field of EM-POCUS and/or medical education, we could not determine how they analyzed our feedback to modify their rating for any given objective. The lack of face-to-face interactions also made it impossible to gauge the time and rigor individual panelists invested in this project. However, the panel response rate of more than 75% across all rounds demonstrated a high level of interest and commitment to the study. The possibility exists that panelist efforts varied greatly, and yet we treated their contributions equally. A second limitation is that the opinion of these 46 panelists may not necessarily reflect that of all the experts in the field. 

## Conclusions

This modified Delphi consensus study recommends the first set of an expert-derived objectives for EM-POCUS curriculum for Nepal. This may guide training programs yet to adopt POCUS education and standards for those programs with existing curricula. As the field of EM-POCUS continues to grow, we anticipate that future iterations of our EM-POCUS applications will require modifications to include more advanced material.

## Conflict of interest

The authors declare no conflict of interest relevant to this work.

## Funding

None

## Author contributions

RS, APS, WB, and AD contributed to the conception and design of the research. RS, APS, SKS, SPS, SB, RH, SM, WB, UH, RD, and RKD contributed to the analysis of the data and formulation of the initial list of objectives. APS, RS, RH, SM, WB, and UH drafted the manuscript. All authors critically revised the manuscript, agreed to be fully accountable for ensuring the integrity and accuracy of the work, and read and approved the final manuscript. 

## Supplementary Material

Supplemental file 1 (S1) CREDES checklist.

Supplemental file 2 (S2) Competency categories and their objectives.
