# Supplementary material for: Delphi Consensus Recommendations for the Development of the Emergency Medicine Point of Care Ultrasound (POCUS) Curriculum in Nepal
Source: POCUS J. 2024 Nov 15;9(2):133–42. doi: 10.24908/pocus.v9i2.17724 (PMC11616984; doi:10.24908/pocus.v9i2.17724)
Supplement: Supplemental file 1 (S1) [file pocusj-09-02-17724-s001.docx]

Supplemental file 1. CREDES checklist

| Guidance on Conducting and Reporting Delphi Studies (CREDES) Checklist | |
| --- | --- |
| **Rationale for Delphi Technique** |  |
| ***Justification.*** The choice of the Delphi technique as a method of systematically collating expert consultation and building consensus needs to be well justified. | Introduction  Page 4-5 |
| **Planning and Design** |  |
| ***Planning and process.*** The Delphi technique is a flexible method and can be adjusted to the respective research aims and purposes. Any modifications should be justified by a rationale and be applied systematically and rigorously | Methods  Page 5-10 |
| ***Definition of consensus.*** Unless not reasonable due to the explorative nature of the study, an a priori criterion for consensus should be defined. This includes a clear and transparent guide for action on (a) how to proceed with certain items or topics in the next survey round, (b) the required threshold to terminate the Delphi process and (c) procedures to be followed when consensus is (not) reached after one or more iterations | Methods  Page7-10 |
| **Study Conduct** |  |
| ***Informational input.*** All material provided to the expert panel at the outset of the project and throughout the Delphi process should be carefully reviewed and piloted in advance in order to examine the effect on experts’ judgements and to prevent bias | Methods  Page No. 6 |
| ***Prevention of bias.*** Researchers need to take measures to avoid directly or indirectly influencing the experts’ judgements. If one or more members of the research team have a conflict of interest, entrusting an independent researcher with the main coordination of the Delphi study is advisable | No conflicts reported  Page No.7 |
| ***Interpretation and processing of results.*** Consensus does not necessarily imply the ‘correct’ answer or judgment; (non)consensus and stable disagreement provide informative insights and highlight differences in perspectives concerning the topic in question | Methods  Page No. 9-10 |
| ***External validation.*** It is recommended to have the final draft of the resulting guidance reviewed and approved by an external board or authority before publication and dissemination | Results  Page No. 11 |
| **Reporting** |  |
| ***Purpose and rationale.*** The purpose of the study should be clearly defined and demonstrate the appropriateness of the use of the Delphi technique as a method to achieve the research aim. A rationale for the choice of the Delphi technique as the most suitable method needs to be provided | Introduction  Page No. 4-5 |
| ***Expert panel.*** Criteria for the selection of experts and transparent information on recruitment of the expert panel | Methods  Page No.5-6 |
| ***Description of the methods.*** The methods employed need to be comprehensible; this includes information on preparatory steps, piloting of material and survey instruments, design of the survey instrument(s), the number and design of survey rounds, methods of data analysis, processing and synthesis of experts’ responses to inform the subsequent survey round and methodological decisions taken by the research team throughout the process | Methods  Page No. 7-10 |
| ***Procedure***. Flow chart to illustrate the stages of the Delphi process, including a preparatory phase, the actual ‘Delphi rounds’, interim steps of data processing and analysis, and concluding steps | Figure 1 |
| ***Definition and attainment of consensus***. It needs to be comprehensible to the reader how consensus was achieved throughout the process, including strategies to deal with non-consensus | Methods  Page No. 9 |
| ***Results.*** Reporting of results for each round separately is highly advisable in order to make the evolving of consensus over the rounds transparent. This includes figures showing the average group response, changes between rounds, as well as any modifications of the survey instrument such as deletion, addition or modification of survey items based on previous rounds | Table  Figure |
| ***Adequacy of conclusions.*** The conclusions should adequately reflect the outcomes of the Delphi study with a view to the scope and applicability of the resulting practice guidance | Discussion |
| ***Publication and dissemination.*** The resulting guidance should be clearly identifiable from the publication, including recommendations for transfer into practice and implementation. A dissemination plan should include endorsement of the guidance by professional associations and health care authorities to facilitate implementation | This publication and  presentation done in GPEMAN intl conference 2024 |
